# Living related hemi-face skin transplant using radial forearm free flap for a xeroderma pigmentosa patient: early outcome

**DOI:** 10.1186/1758-3284-2-18

**Published:** 2010-07-13

**Authors:** Ayman Amin, Mahmoud Bassiouny, Kareem Sallam, Galal Ghally, Hanaa El-Karaksy, Alaa El-Haddad

**Affiliations:** 1 Surgical oncology department, national cancer instituite, Cairo, Egypt; 2 Pediatrics department, kasr El-eini school of medicine, Cairo, Egypt; 3 Paediaric oncology department, national cancer instituite, Cairo, Egypt

## Abstract

**Introduction:**

Xeroderma pigmentosa (XP) is a hereditary disease characterized by deficient repair of DNA damage that occurred on exposure of the skin to ultraviolet irradiation. The affected children have a propensity to develop multiple skin cancers mainly in the face and eventually die before the age of 20.

**Hypothesis:**

Allograft replacement of facial skin by a healthy skin from normal person might decrease the incidence of skin cancer development, the number of surgical procedures, and eventually might improve the survival of these miserable patients.

**Methods:**

As Cadaveric organs are unavailable in our country. After approval from the ethical committee, confirmed agreement of the donor and the patient's guardian, a radial forearm free flap was transplanted from an ABO compatible mother to her 5 year old daughter with XP. The mother had an older daughter died from the same disease at the age of 14. The flap replaced skin of the hemi face that developed precancerous lesions. The girl was kept on adjusted doses of immunosuppressive drugs.

**Results:**

The flap survived, wounds healed uneventfully. The flap developed a reddish spot one and half month following transplant where baseline skin biopsy was taken. In the fifth months the girl presented with bad non salvageable rejection that ended up loosing the flap. On long term follow up, the girl started to develop skin lesion on the virgin half of the face. Our early cosmetic result replacing half of the facial skin was very promising. In addition the girl did not develop skin lesions in the operated site.

**Conclusion:**

Our early cosmetic result was very promising. In addition to this, the girl did not develop skin lesions in the operated side of the face

## Introduction

Xeroderma pigmentosa (XP) is an autosomal recessive genetic disorder that makes the DNA of the skin unable to repair the continuous damage inflicted on it by the Ultra Violet (UV) rays present in sun rays [1]. A dominant form of XP was described in a Scottish girl; these patients have a mild clinical course [2]. The disease is characterized by photosensitivity, pigmentary changes, premature skin ageing, neoplasia and abnormal DNA repair. Some patients also have neurological complications. Affected individuals are 1000 times more prone to UV induced skin cancer than unaffected ones and over 90% of affected individuals will develop skin malignancy before the end of their second decade [3]. Development of multiple recurrent skin malignancy is the eventual outcome of this DNA repair failure which world-wide kills two thirds of the affected subjects before the age of 20. Death follows a lengthy devastating illness for both the patient and his family where malignant lesions can develop as early as the third or fourth year [2]. During their life span they get exposed to repeated surgical resection of newly-developed skin cancers. Surgical resection ranges from simple excision and skin closure to heroic cranio-facial resection and free flap reconstruction.

In a desperate attempt to prevent malignant transformation, affected individuals have to be kept away from sun light through out there life. There is no definitive treatment to the disorder up to the moment. Current interventions aim at preventing, or better say, delaying the occurrence of malignancy using both medical and surgical approaches, none of them is truly successful. Replacement of the skin of the face represents one of the approaches. It has been tried using autologous skin grafts, but again skin grafts (that carries the same genetic disorder) developed malignancy when transferred to the face.

Hypothesis: If the skin of the face can be replaced by skin from a healthy individual (not carrying the genetic disorder), it should not develop malignancy when exposed to the UV rays. This might decrease the incidence of skin cancer development, the number of surgical procedures, improve the quality of life and eventually the survival of those miserable patients.

## Materials and Methods

As cadaveric organs are unavailable in our country, a sensate radial forearm free-flap will be harvested from ABO compatible and HLA typed living related donor. A three-staged procedure was planned:

Stage one: Full thickness Excision of the skin of one side of the face and replacing it immediately by a sensate free vascularized fasciocutaneous radial forearm flap harvested from her healthy donor. Part of the face is to be covered by split thickness graft again from the donor to assess any difference in performance between the fasciocutaneous flap and split thickness grafts when used as allograft to guide future procedures. The donor site is to be closed by an auotologous partial thickness skin graft.

Stage two: Immune suppression and postoperative care.

Immunosuppressive regimen:

1- Corticosteroids:

• Methylprednisolone: 10 mg/kg/day, single daily dose was started 2 days pre-operative until day 3 post-operative

• Methylprednisolone: 5 mg/kg/day, single daily dose from day 4-day 6 post-operative

• Prednisone 2 mg/kg/day divided in 3 daily doses started from day 7 and tapered gradually over the next 3 months and was discontinued on day 90 post-operative

• Monitoring for steroids side effects was carried out by 6 hourly measurement of blood pressure while the patient was hospitalized and every visit thereafter. Blood sugar was also closely monitored during hospital stay.

2- Cyclosporine (Neoral): was started at a dose of 5 mg/kg/day 2 days before surgery and monitored thereafter by trough level around 150 ng/ml. Monitoring of trough level was done twice weekly at the start until the desired level was achieved and monthly thereafter. Kidney functions were monitored twice weekly during hospitalization and on every subsequent visit.

3- Mycofenolate mofetil (Cellcept): was started on day 1 post-operative, 250 mg capsule was given twice per day. Patient was monitored for myelosuppression by twice weekly complete blood counts in the first 3 weeks and monthly thereafter.

Stage three: after ensuring successful technical and immunologic outcome, the rest of the face is to be subjected again to excision of the native skin and coverage by either tissue expansion of the transplanted skin or by another transplant.

Should any unfavorable outcome be encountered as regards the viability of the transplanted skin, it is to be replaced by autologous split thickness graft which is the eventual end point for these patients if left untreated until development of malignancy.

## Consent

After approval from the ethical committee of the National Cancer Institute. Fully informed counseled consent was taken from the patient's parents. This included discussion of alternative treatment options, an emphasis about the fact that the procedure is a new procedure for that kind of pathology, possible complications of the proposed procedure, the need for postoperative immune suppression and its potential complications. 'Written informed consent was obtained from the patient for publication of this case report and accompanying images. A copy of the written consent is available for review by the Editor-in-Chief of this journal. We performed the first case of living-related facial skin transplant from a mother to her 5-year old daughter with XP who started development of precancerous skin lesion. The mother had an older daughter who died from the same disease at the age of 14. Surgery was done on May 28, 2008.

### Postoperative management

The patient was isolated in a surgical ICU, the viability of the transplanted skin was monitored by visual observation and palpation of temperature as well as by hand-held Doppler

## Results

**Figure 1:**
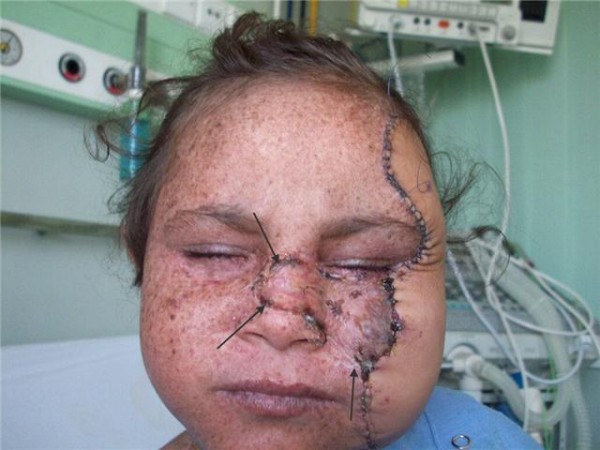
**Early postoperative outcome front view with the area covered by split thickness skin graft defined by arrows**.

**Figure 2:**
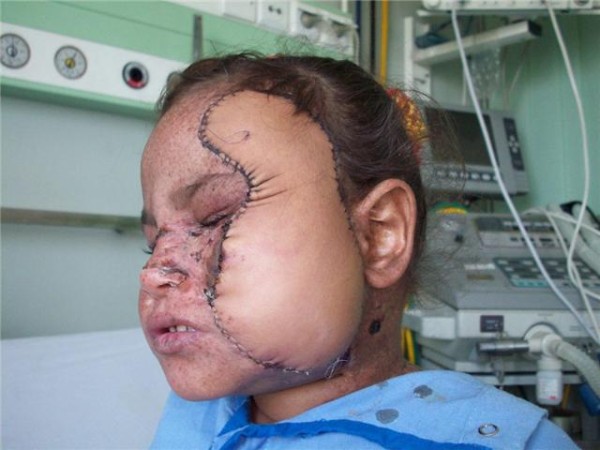
**Side view showing the healthy radial forearm flap**.

**Figure 3:**
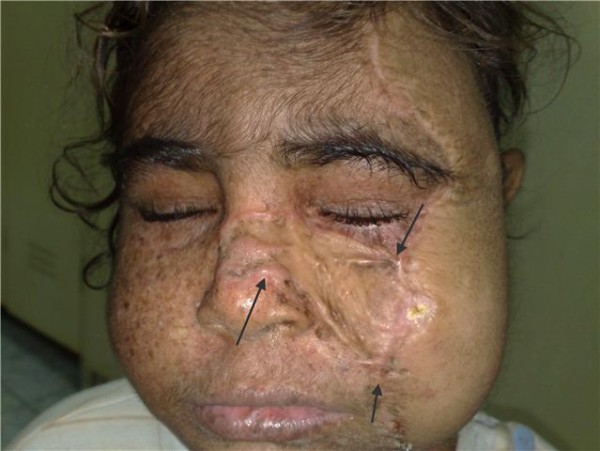
**Cosmetic outcome after the flap has remodeled to the face of the patient**.

Surgical pathology of the excised skin showed foci of marked dysplasia and carcinoma in situ lesions. The flap survived the early postoperative period. The wounds healed uneventfully and the patient was discharged on a weekly outpatient visit for follow up (Figures 1, 2, 3). Immune suppression was done as planned as given in detail in the methods portion.

**Figure 4:**
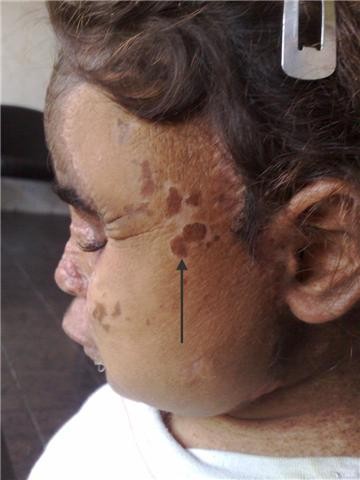
**Spots marked by an arrow denoted early rejection**.

**Figure 5:**
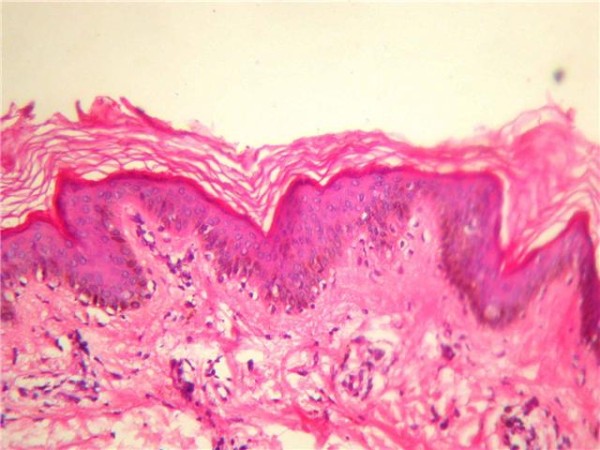
**Base line skin biopsy showing lymphocytic infiltration**.

**Figure 6:**
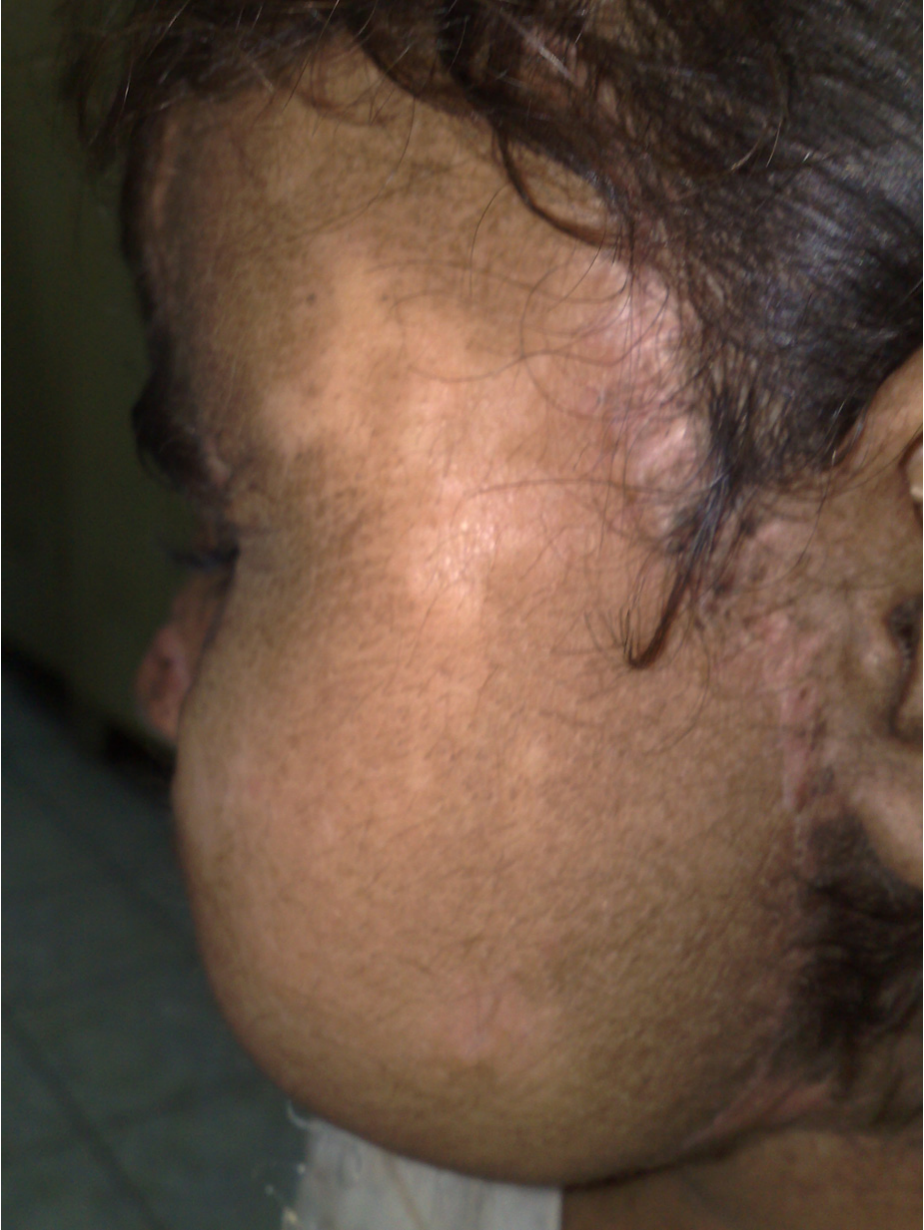
**Spots have faded away**.

One and half month following the transplant: The girl developed a reddish spot on the flap where a baseline skin biopsy was taken that revealed minimal immunologic reaction, and this spot faded away spontaneously (Figures 4, 5, 6).

**Figure 7:**
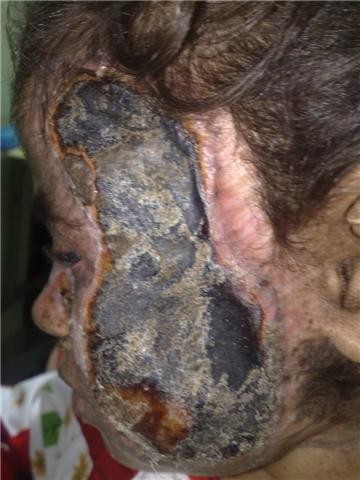
**Irreversible graft rejection**.

**Figure 8:**
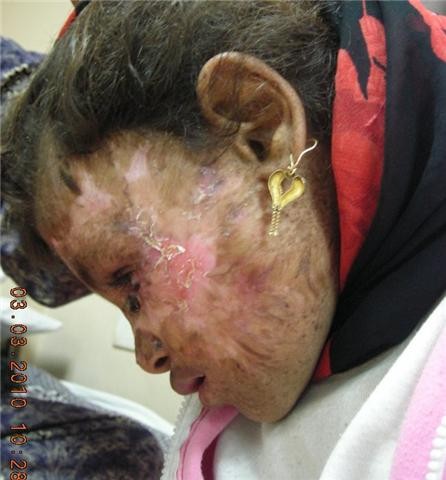
**complete healing by spontaneous granulation 5 months after removal of the flap**.

**Figure 9:**
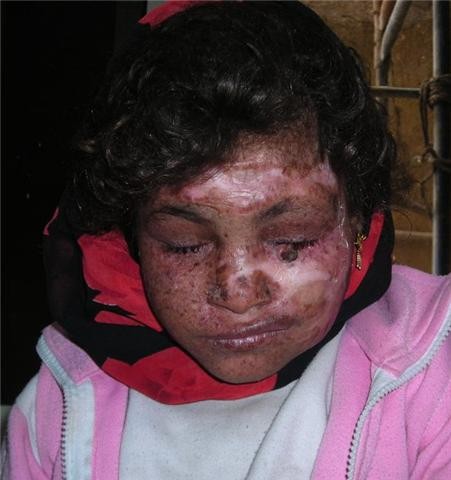
**18 months later showing the cosmetic outcome after healing with secondary intention following debridement of the rejected flap (front view)**.

**Figure 10:**
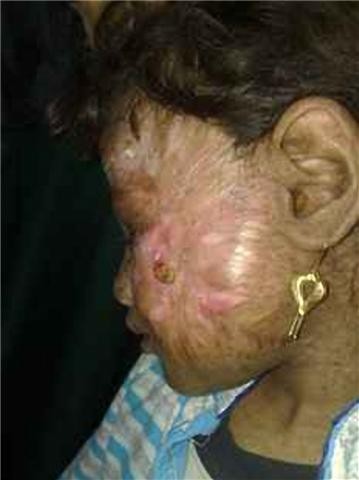
**Lateral view**.

**Figure 11:**
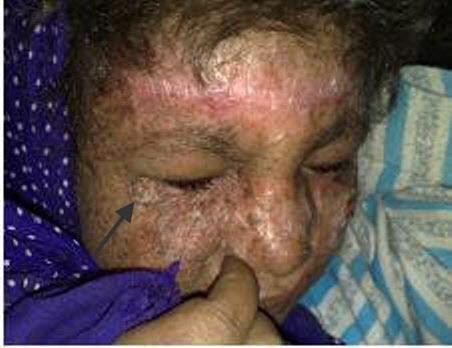
**Ten months post transplant where the patient started to develop skin lesions in the contra lateral side (marked by an arrow)**. Worth noting that the side operated upon and left to heal by secondary intention did not develop any new lesions.

**Figure 12:**
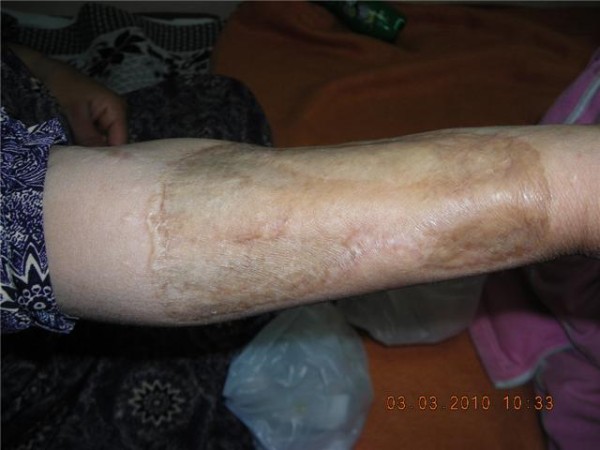
**Late Postoperative follows up of the donor site in the mother's forearm; covered by a split thickness graft**.

Two months post transplant: The girl was doing fine, apart from hirsutism form the cyclosporine. The girl has missed the follow up for 3 weeks before this visit and presented, in the fifth month, with clinical evidence of graft rejection. The trough level of Cyclosporine at presentation was below the target level. Interestingly enough, the skin graft was not affected with the rejection process. A trial of salvaging the flap by corticosteroids and manipulating the immunosuppressants was unsuccessful, so the flap was removed, immunosuppressants were discontinued and the face was left to granulate (Figures 7, 8, 9, 10). For obvious reasons, the third stage of the planned procedure (tissue expansion) was aborted. By the 10^th ^month Postoperative, the patient started to develop malignant lesions in the non-operated side of her face (Figure 11). The donor site in the mother that was closed by a partial thickness skin graft healed uneventfully as well (Figure 12).

## Discussion

Xeroderma pigmentosa is a devastating morbidity for the patient and the family as well as the caring medical team. The author's previous experience with this lethal devastation inspired executing the idea with any prospect of success. The mother having witnessed the life and death of her previously affected offspring, unsurprisingly, accepted with enthusiasm donating her skin. Attempts to resurface their faces have been tried using autologous skin that ended up developing malignant lesions as it still carries the same genetic defect. To the best of the author's knowledge, this is the first attempt of allotransplantation for patients with Xeroderma pigmantosa.

There were two issues as regards timing in this procedure. First, is whether to do the transplantation before or after development of malignant lesion? Second, is whether to do the skin expansion before or after transplantation i.e. in the arm of the mother or in the face of the patient? Prophylactic excision was chosen in this case to avoid the use of immunosuppressants in the face of a coexisting invasive malignancy. It might seem logical and tempting to expand the skin in the arm of the mother to allow harvesting ample tissue with the expander in a rather convenient anatomical site. However, tissue expansion was deferred to a post-transplant stage. The reason was that transferring ample skin would have simply meant excision of ample facial skin as well from the patient. Should removal of the graft prove to be necessary for any reason; a large raw area would have resulted in such a case.

The rationale behind the attempt was that the development of skin malignancy is almost certain in these patients. Standard treatment is excision and split thickness grafting. Therefore, loosing the graft and resurfacing by autologous split thickness graft is not too far from this end point if the patient was left untreated. Immune suppression in a patient well known to be prone to malignancy was an issue of concern. Experience from long term follow up of renal transplant patients pointed to Immune suppression as a risk factor for developing malignancy with an 18-fold rise in risk for developing squamous carcinoma in these patients [4]. This was not enough for the authors to abort the idea as xeroderma pigmentosa patients, when left untreated, they systematically develop malignancy and usually die in the second decade of life, so they simply can not be rendered "more prone" to malignancy. They are already at the height of susceptibility. Given this fact, the authors chose not to loose the grip on the idea and they chose to see whether this theoretical (but certainly relevant) risk will really set in on clinical grounds or not, as this risk has not been assessed clinically in this particular subset of patients. In addition, hepatologists extended the indications for liver transplantation to include some patients with hepatocellular carcinoma. They do receive immunosuppressants in the immediate postoperative period. Whether Immune suppression accelerates the malignant process in this subset of patients or not, reports are conflicting and it seems that the best answer to this question is the answer given Schwartz et al; "we do not know" [5].

We believe that the way out of this morbidity is either prevention by genetic counseling and prenatal diagnosis or by gene therapy for those whom it happened that they are born with the defect. Genetic counseling and prenatal diagnosis are currently available in clinical practice but gene therapy is still investigational. Various methods of correcting the defects in xeroderma pigmentosum have been attempted in vitro and in animal studies using viral vectors (adenoviruses and retroviruses) carrying the gene replacement products. Ex vivo skin gene therapy, which refers to grafting skin that has the genetic defect corrected may be useful in xeroderma pigmentosum in the future [6]. A new approach is to repair DNA damage after UV exposure. This can be accomplished by delivery of a DNA repair enzyme into the skin by means of specially-engineered liposomes [7]. Employing this therapy over a period of 1 year, Yarosh et al [8] demonstrated a reduction in the onset of actinic keratosis and Basal cell carcinoma. Oral retinoids have been shown to decrease the incidence of skin cancer in patients with xeroderma pigmentosum. This therapy is limited by dose-related irreversible calcification of ligaments and tendons.

As mentioned earlier, Skin excision and grafting by an auto graft (total resurfacing) ended up by malignancy developing in the grafted skin when it was transplanted to sun exposed areas. Chemical peeling and dermabrasion represent a less invasive method of resurfacing with reports of a disease free period up to 4 years (free from malignant lesions). However, protection from sun is still mandatory after the procedure [9]. The attempt described in this article can be looked at as an evolution of the total resurfacing approach with the next step seems to be cadaveric total face transplantation. Clinical attempts of composite tissue allotransplantation have been described between identical twins [10,11]. When the donor is not an identical twin, these reconstructive procedures for non life-threatening indications remain rare due to adverse effects of the associated lifelong immunosuppressive therapy [12]. Face transplantation is emerging as a solution to traumatic events with the first report of a dynamic hemi face transplant following a dog bite performed in France [13]. There are three dissimilarities, however, between the case performed in France and this report. These are; the indication of the procedure, the type of tissue transplanted and lastly the regimen of immune suppression. The indication in our report was prevention of malignancy while in the patient reported in France the indication was reconstruction of a lower face amputated by a dog bite. The graft transplanted in France was a composite graft containing skin, muscles and mucous membrane with their nerve and blood supply harvested from a brain-dead lady in her forties. As mentioned earlier, ethical committees in our country have not yet approved brain-dead patients as donors which made a living-related donor the author's only option which consequently dictated the type of tissue that can be harvested. As regards immune suppression regimen; the author's choice of drugs (described earlier) in the current case was, in part, influenced by the available fund allocated for the attempt. This immunosuppressive regimen was modified from the regimen used by members of the same team who work in the living related liver transplantation program at Cairo University. Because skin is a mixed tissue that is liable to more rejection than the liver, the regimen used a dose of steroids that was higher than that used for liver transplant recipients and was even started earlier pre-operatively. Also, a combination therapy of cyclosporine and mycofenolate mofetil was used from the start. Liver transplant recipients receive monotherapy with tacrolimus (after steroids are discontinued) and mycofenolate is added as rescue therapy if the recipient experiences acute rejections.

At the time of this skin transplant for the xeroderma pigmentosa case, m-TOR inhibitors (sirolimus and everloimus) were unavailable; which is not the condition now. During the last year they were used in liver transplant recipients who experience repeated acute rejections not controlled by proper doses of tacrolimus. The pediatric hepatologists in the liver transplant team have also used sirolimus in one case of chronic rejection (a rare condition) in a child and chronic rejection was successfully controlled.

In the case performed in France, the Immunosuppressive treatment was with thymoglobulin, tacrolimus, mycophenolate mofetil, and prednisone. Two infusions of donor bone-marrow cells were given. They described 2 episodes of rejection in 18 months follow up that were reversed [14]. Another case was reported from China were, in 2006, Guo et al performed a partial face transplant to reconstruct a face of a man who sustained a bear bite. They used Quadraple immunomodulatory therapy containing tacrolimus, mycophenolate mofetil, corticosteroids, and humanized IL-2 receptor monoclonal antibody, and in two years follow up, they reported 3 episodes of rejection controlled by immune suppressive therapy adjustmen [15]. Finally, a report of near total face transplantation came from Cleveland in December, 2008. The patient was a lady who sustained a shotgun [16].

## Conclusion

Although we cannot comment on long term results due to graft rejection, our early cosmetic result was very promising. In addition to this, the girl did not develop skin lesions in the operated site but developed one in the virgin hemi-face. We believe that if we succeeded to highlight the problem of these kids and ignite more research in this direction, then we already achieved our goal.

## Competing interests

The authors declare that they have no competing interests.

## Authors' contributions

AA has provided the idea and was the main surgeon involved in the care of the patient at different stages. MB was a main operating surgeon, KS is a surgeon who assisted the main author, GG has provided postoperative surgical care and follow up of immunesuppression regimen. HE and AE carried out the perioperative and maintenance immunosuppression regimen. All authors read and approved the final manuscript.
